# Bilateral simultaneous complete quadriceps rupture following chronic symptomatic tendinopathy: a case report

**DOI:** 10.4076/1752-1947-3-9031

**Published:** 2009-09-08

**Authors:** Buchi Arumilli, Foley Adeyemo, Richard Samarji

**Affiliations:** 1 Manchester Royal Infirmary, Oxford Road, Manchester, M13 9WL, UK

## Abstract

**Introduction:**

Quadriceps rupture is a disabling injury mostly seen in men over 40 years of age. Bilateral quadriceps rupture is a rare injury that is often secondary to predisposing medical conditions. Ultrasound is a cheap and reliable tool for diagnosis but is operator dependent. Thus, magnetic resonance imaging is the preferred method of investigation despite its cost and availability. Prompt diagnosis and early surgical repair are needed for an optimal end result.

**Case presentation:**

We report the case of a healthy 54-year-old Caucasian male farmer who presented with bilateral simultaneous complete quadriceps rupture, which was managed surgically and he was followed up for three years. He was previously under our care for enthesopathy of the quadriceps on both sides. We believe that chronic enthesopathy of the superior pole of patella made his quadriceps susceptible to complete rupture on eccentric loading.

**Conclusion:**

Only a few cases of bilateral simultaneous complete quadriceps rupture in patients with symptomatic enthesopathy have been previously reported. We stress the importance of warning patients of the risk of developing complete tendon rupture when they present with an enthesopathy around the knee.

## Introduction

Quadriceps tendon rupture is a serious and disabling injury that can occur as a result of a direct or, more commonly, an indirect mechanism. Bilateral quadriceps tendon ruptures can occur spontaneously or following minimal trauma. Approximately 30% of bilateral simultaneous quadriceps rupture cases happen to patients with underlying medical predispositions [1]. The common predisposing medical conditions related to quadriceps rupture are diabetes, chronic renal failure and hyperparathyroidism. Crystal arthropathy, inflammatory arthritis and obesity are also proven to be predisposing conditions [2]. Taking drugs such as quinolones and undergoing systemic steroid therapy are other underlying causes. The literature on bilateral quadriceps rupture also shows systemic steroid use, systemic lupus erythematosus, pseudogout and occupational trauma as the aetiology of this condition [2]. Chronic enthesopathy of the quadriceps can present as an anterior knee pain and the superior pole of the patella is the site of pathology in 25% of patients [1]. We present a case of bilateral simultaneous quadriceps rupture in a patient who was under our care for chronic tendinopathy at the superior pole of the patella of both knees.

## Case presentation

A 54-year-old Caucasian male farmer was seen in our department following a referral from his general practitioner for a non-traumatic bilateral anterior knee pain. Plain radiographs revealed a phase II chronic tendinopathy of the superior pole of patella on both sides of his knee. This was attributed to his frequent squatting and kneeling on the farm. He was managed with physiotherapy and he improved symptomatically. Neither his general practitioner nor our department gave him steroid injections.

Two years later, he presented to our Accident and Emergency Department with sudden bilateral knee pain following a tumble on the farm. He was unable to get up and stand on his feet and was brought in by an ambulance. Further enquiry revealed that the injury happened when both his feet got stuck in the mud on a wet day and both his knees buckled under his weight.

**Figure 1:**
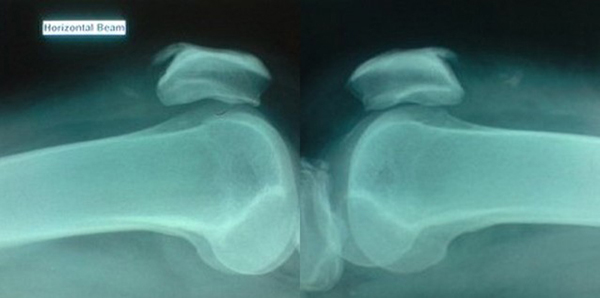
**Lateral radiographs of both knees**. The initial X-rays revealed only the patellar spur at the superior pole of the patella and some calcification in the quadriceps tendon.

**Figure 2:**
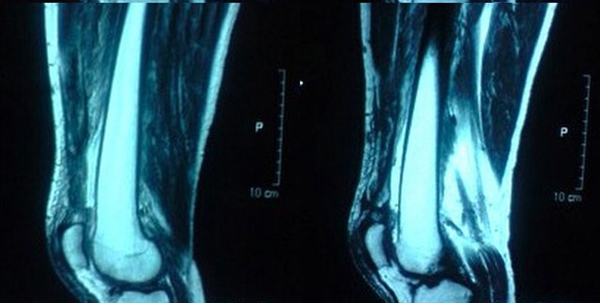
**Magnetic resonance images of the knees**. The magnetic resonance scan revealed disruption of the quadriceps at a level of 1.5 cm from the insertion into the patella on both sides, right more extensive than left. There was thickening of the quadriceps on both sides.

Clinically active knee flexion was possible without any ability to extend. Moderate hemarthrosis on both of his knees was present. Initial X-rays revealed only the bilateral patellar spurs with calcific shadows in the quadriceps tendon (Figure 1). A definitive gap was not palpable in the suprapatellar region but he was tender over the area. A magnetic resonance imaging scan done immediately on both his knees (Figure 2) revealed a complete tear of the quadriceps at approximately the same level (1.5 cm from the insertion) of the tendon (midsubstance) rather than the osteotendinous junction. The transverse sections of the patella bore significant lateral tilt on either side, with the left side showing a more pronounced tilt, indicating bilateral torn retinaculae. He was investigated for any underlying predisposing causes. His bone profile, renal profile, blood glucose, serum uric acid and lipid profile were normal. He was HLA B27 negative and his body mass index was 29.

**Figure 3:**
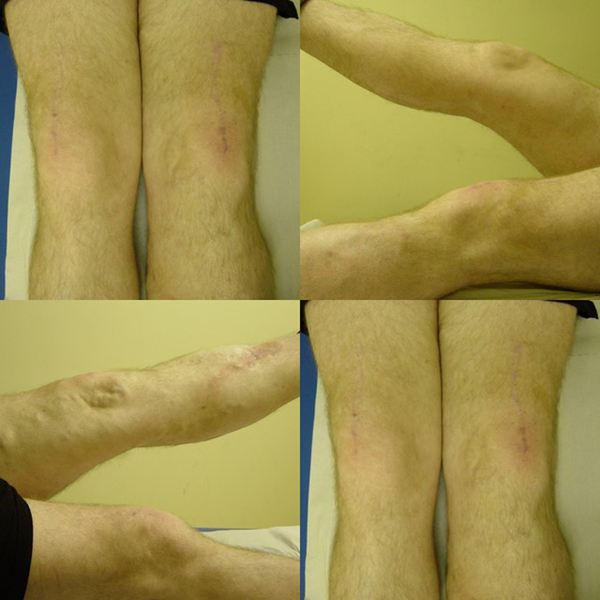
**Follow-up pictures at three years after surgical repair**. The well-healed operation sites on both knees. The active extension (straight leg raise) on both sides showing no significant extensor lags.

He was operated upon and underwent a tendon-to-bone repair of the quadriceps on both sides because the distal stump of his tendon appeared degenerate. It was also discovered that the retinaculae on both of his knees were torn. Apart from moderate degeneration of the tendon no striking abnormalities were detected intraoperatively. Postoperatively, both knees were splinted in extension for three weeks and he later started on a range of movement exercises of up to 90 degrees of flexion. He was allowed to fully bear weight six weeks after his surgery. Three years after the surgery, he had an excellent result with full active extension (Figure 3) and a full range of knee movement. His Tegner-Lysholm score three years after the operation was 99, which is categorised as "excellent".

## Discussion

The first bilateral simultaneous quadriceps rupture was reported by Steiner and Plamer in 1949. Since then, just over 100 such cases with different etiopathologies have been reported in the English and German literature [2]. Diagnostic delay is quite common especially in bilateral ruptures, as they can be mistaken for stroke, rheumatoid arthritis, disc prolapse, neuropathy or even a psychiatric disorder [2].

The quadriceps tendon is an inherently very strong structure that is extremely resistant to heavy load. McMaster, in his animal studies, postulated that about 50-75% of the fibres of the quadriceps need to be severed before it will rupture totally under a physiological load [1]. The common mechanisms of trauma include a stumble, a simple fall, falling from the stairs or from a height. The ratio of ligamentum patellae and quadriceps tendon forces is a function of the knee flexion [3], hence quadriceps ruptures tend to occur commonly when the knee is flexed more than 60 degrees. At about 30 degrees of knee flexion, the force through the extensor mechanism reached an average of 3000 N, along with the highest lateral forces on the patella [4]. Most of the injuries occur during an eccentric contraction of the quadriceps against the body weight, when a significantly higher force is generated [1].

Degenerative changes commonly occur in the tendons as the ageing process causes their architecture to change. However, quadriceps tendon rupture is rare even among older people [1]. Thus, other underlying factors, and not age, predispose the tendon to rupture.

An array of conditions has been reported to predispose the rupture by either changing the tendon ultrastructure or affecting the vascularity to the tendon [5]. Thirty percent of bilateral ruptures are spontaneous [2]. Pre-existing degeneration has been implicated as a risk factor in acute tendon rupture.

The tensile strength of a tendon is related to its thickness and collagen content. An objective measure quoted in one study shows that a quadriceps tendon with a cross-sectional area of 65 mm^2^ has a tensile stress value of 37 N/mm^2^[6]. Degeneration as a common histological finding in spontaneous tendon ruptures has been reported commonly in the achilles tendon. Tendons respond to repetitive overload with either inflammation of their sheath or degeneration, or even both. Mucoid degeneration is more common in quadriceps and patellar tendons. Tendinosis can be more often clinically silent. Its manifestation may be a rupture, although it may also co-exist with symptomatic paratendinopathy [5].

Ultrasound examination, rather than plain radiograph, is more sensitive in demonstrating the full extent of the tendinopathy. It is also a cheap, easy and reliable way to diagnose tendon ruptures, whether partial or complete. However, ultrasound examination is still operator dependent.

A quadriceps tendon thickness of >6.1 mm, a superior pole of patella erosion, the patellar enthesophytes and intratendinous calcification are all signs of chronic tendinopathy [7]. Hardy *et al.* reported that 79% of patients diagnosed with quadriceps rupture had a patellar spur on lateral knee radiographs [8]. A report of consequent bilateral quadriceps rupture following trivial trauma was reported in a middle-aged man who had a chronic patellar maltracking postulating patellofemoral arthritis as the predisposition [9]. Calcific tendonitis of the quadriceps was reported along with a quadriceps tear with liquefied calcific collection in the tendon on exploration [10].

Our patient had established changes such as the thickening of his quadriceps tendon, patellar spurs and intratendinous calcifications on both sides of his knee, about two to three years before complete rupture occurred. His farming occupation was attributed as the cause of the pathology due to his frequent squatting and prolonged knee flexion. This could have contributed to the rupture due to the local ischemic changes produced in the tendon.

Our patient did not undergo ultrasonography and hence we did not know the extent of the degeneration of the soft tissue. The rupture was at the intratendinous part of the quadriceps on both sides. His quadriceps was ruptured at the same level, which is an uncommon occurrence.

In a meta-analysis of 52 quadriceps ruptures, osteotendinous junction was the most common site of failure [2]. A range of medical conditions have been associated with quadriceps ruptures, especially those that are bilateral [2]. Bilateral complete quadriceps ruptures that are simultaneous and intratendinous in patients with previous chronic symptomatic tendinopathy are rare. Although chronic enthesopathy cannot be cited as the only cause in our patient, a combination of tendinosis, ischaemia and trauma might have caused the final injury.

We think an objective assessment (bony and soft tissue changes) of the degree of degeneration or even histopathological status of the tendon needs to be developed in order to identify patients most at risk of ruptures or at least warn them of this possibly disabling complication.

## Conclusion

The quadriceps is a strong structure but may be weakened by many systemic and local factors. Symptomatic enthesopathy is rarely the only cause of bilateral simultaneous complete quadriceps rupture. Objective grading of structural changes in chronic tendinopathy in all patients will identify those most at risk of rupture.

## Competing interests

The authors declare that they have no competing interests.

## Consent

Written informed consent was obtained from the patient for publication of this report and any accompanying images. A copy of the written consent is available for review by the Editor-in-Chief of this journal.

## Authors' contributions

BA carried out the literature search and wrote the manuscript. FA compiled the references and images. RS performed the surgery on the patient and initiated the making of this case report. All authors read and approved the final manuscript.
